# 5-(2-Meth­oxy­phen­yl)-1,3,4-thia­diazol-2-yl 2-meth­oxy­benzoate hemihydrate

**DOI:** 10.1107/S1600536811010373

**Published:** 2011-04-07

**Authors:** Jin-hua Yao, Bing Guo, Kang An, Jian-ning Guan

**Affiliations:** a Department of Applied Chemistry, College of Science, Nanjing University of Technology, No.5 Xinmofan Road, Nanjing, Nanjing 210009, People’s Republic of China

## Abstract

In the title compound, C_17_H_14_N_2_O_4_S·0.5H_2_O, the mol­ecule, with the exception of the two meth­oxy­phenyl groups, is nearly planar with an r.m.s. deviation of 0.0305 Å. The two 2-meth­oxy­phenyl rings make dihedral angles of 4.1 (3) and 2.3 (3)° with the thia­diazole ring. In the crystal, inter­molecular C—H⋯O and O—H⋯N hydrogen bonds link the mol­ecules.

## Related literature

For general background to 1,3,4-thia­diazole derivatives, see: Matysiak & Opolski (2006 ▶). Alireza *et al.* (2005 ▶). Wang *et al.* (1999 ▶). For bond-length data, see: Allen *et al.* (1987 ▶). For the synthesis, see: Kurzer (1971 ▶).
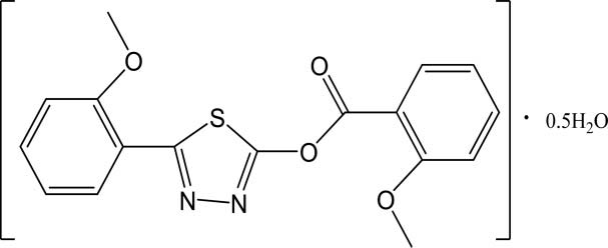


## Experimental

### 

#### Crystal data

C_17_H_14_N_2_O_4_S·0.5H_2_O
*M* *_r_* = 356.37
Monoclinic, 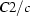

*a* = 29.858 (6) Å
*b* = 14.542 (3) Å
*c* = 7.6710 (15) Å
β = 95.19 (3)°
*V* = 3317.1 (12) Å^3^
*Z* = 8
Mo *K*α radiation
μ = 0.22 mm^−1^
*T* = 293 K
0.30 × 0.20 × 0.10 mm

#### Data collection

Enraf–Nonius CAD-4 diffractometer
Absorption correction: ψ scan (North *et al.*, 1968 ▶) *T*_min_ = 0.936, *T*_max_ = 0.978
3108 measured reflections
3050 independent reflections
1881 reflections with *I* > 2σ(*I*)
*R*_int_ = 0.025
3 standard reflections every 200 reflections  intensity decay: 1%

#### Refinement

*R*[*F* ^2^ > 2σ(*F* ^2^)] = 0.063
*wR*(*F* ^2^) = 0.176
*S* = 1.00
3050 reflections
228 parameters
H atoms treated by a mixture of independent and constrained refinement
Δρ_max_ = 0.41 e Å^−3^
Δρ_min_ = −0.28 e Å^−3^

### 

Data collection: *CAD-4 EXPRESS* (Enraf–Nonius, 1994) ▶; cell refinement: *CAD-4 EXPRESS* ▶; data reduction: *XCAD4* (Harms & Wocadlo, 1995 ▶); program(s) used to solve structure: *SHELXS97* (Sheldrick, 2008 ▶); program(s) used to refine structure: *SHELXL97* (Sheldrick, 2008 ▶); molecular graphics: *SHELXTL* (Sheldrick, 2008 ▶); software used to prepare material for publication: *SHELXL97*.

## Supplementary Material

Crystal structure: contains datablocks global, I. DOI: 10.1107/S1600536811010373/bq2283sup1.cif

Structure factors: contains datablocks I. DOI: 10.1107/S1600536811010373/bq2283Isup2.hkl

Additional supplementary materials:  crystallographic information; 3D view; checkCIF report

## Figures and Tables

**Table 1:** Hydrogen-bond geometry (Å, °)

*D*—H⋯*A*	*D*—H	H⋯*A*	*D*⋯*A*	*D*—H⋯*A*
O1*W*—H1*W*⋯N2^i^	1.05 (9)	1.81 (9)	2.821 (4)	159 (7)
C11—H11⋯O3^ii^	0.93	2.50	3.324 (4)	148

Symmetry codes: (i) 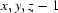; (ii) 
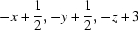
.

**Fig. 1.**
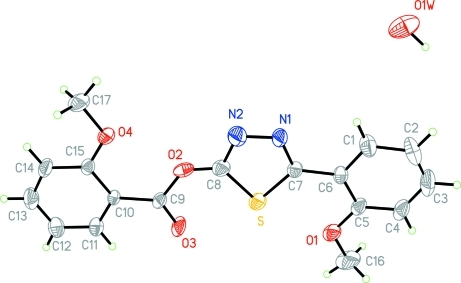
A view of the molecular structure of (I). Displacement ellipsoids are drawn at the 50% probability level.

**Fig. 2.**
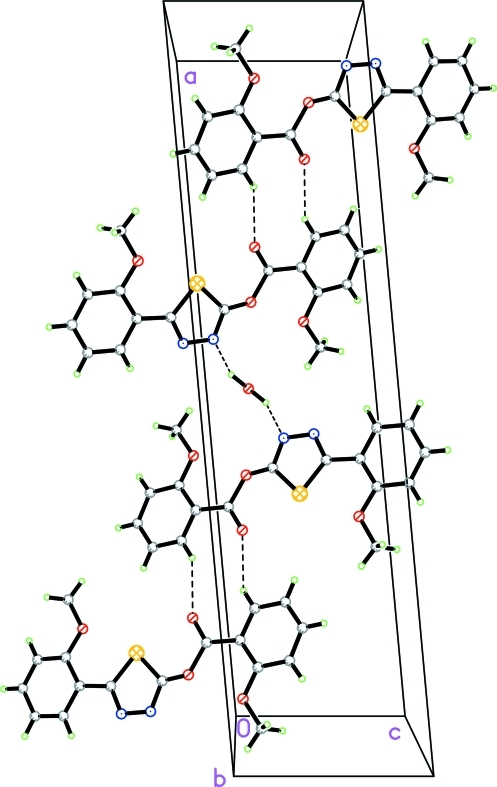
A packing diagram for (I). Dashed lines indicate intermolecular C—H···O and O—H···N hydrogen bonds.

## supplementary crystallographic information

### Comment

1,3,4-Thiadiazole derivatives are of great interest because of their chemical and pharmaceutical properties. Some derivatives play a key role in preparing intermediate for anticarcinogen. Recently new derivatives with 1,3,4-thiadiazole nucleus have been synthesized and evaluated for their antiproliferative effect *in vitro* against the cells of various human tumor cell lines (Matysiak & Opolski, 2006). Some derivatives have effective antibacterial activity. They are of great potential value for killing bacteria (Alireza *et al.* 2005). In addition, this kind of compounds are known to exhibit diverse biological effects, such as insecticidal activity (Wang *et al.* 1999).

Herein we report on the crystal structure of the titled compound, (I). The molecular structure of (I) is shown in Fig. 1. The bond lengths (Allen *et al.* 1987) and angles are within normal ranges. In this structure, there are three rings, ring A (C1/C2/C3/C4/C5/C6), ring B (N1/C7/S/C8/N2) and ring C (C10/C11/C12/C13/C14/C15), all of which are almost planar. Ring B(N1/C7/S/C8/N2) is a planar five-membered ring and the mean deviation from plane is 0.0020 Å. The dihedral angle between ring A and ring B is 4.1 (3)°, ring B and ring C is 2.3 (3)°. In the crystal structure, intermolecular C11—H11···O3 and O1W—H1W···N2 hydrogen bonds (Table 1.) link the molecules to form network structure (Fig. 2), in which they may be effective for the stabilization of the structure.

### Experimental

3-Methoxy-phthalic anhydride(8 mmol) and 2-(2-methoxyphenyl)-5-hydroxy-1,3,4-thiadiazol(8 mmol) were added in ethanol(50 ml) (Kurzer, 1971). The mixture was refluxed for 5 h. Reactions were monitored by thin-layer chromatography (TLC) with visualization by ultraviolet light and then the solvent was totally evaporated. Then the white power was obtained. The solid was recrystallized from tetrahydrofuran to give the compound (I) (m.p. 520 K). Crystals of (I) suitable for X-ray diffraction were obtained by slow evaporation of a mixed solution of chloroform and tetrahydrofuran.

### Refinement

All H atoms were positioned geometrically, with C—H = 0.96 and 0.93 Å for methyl and aromatic H atoms, respectively and constrained to ride on their parent atoms with *U*_iso_(H) = *xU*_eq_(C), where *x* = 1.5 for methyl H atoms and *x* =1. 2 for all other H atoms.

### Crystal data

**Table:**

C_17_H_14_N_2_O_4_S·0.5H_2_O	*F*(000) = 1464
*M**_r_* = 356.37	*D*_x_ = 1.407 Mg m^−^^3^
Monoclinic, *C*2/*c*	Melting point: 520 K
Hall symbol: -C 2yc	Mo *K*α radiation, λ = 0.71073 Å
*a* = 29.858 (6) Å	Cell parameters from 25 reflections
*b* = 14.542 (3) Å	θ = 9–13°
*c* = 7.6710 (15) Å	µ = 0.22 mm^−^^1^
β = 95.19 (3)°	*T* = 293 K
*V* = 3317.1 (12) Å^3^	Block, colorless
*Z* = 8	0.30 × 0.20 × 0.10 mm

### Data collection

**Table:**

Enraf–Nonius CAD-4 diffractometer	1881 reflections with *I* > 2σ(*I*)
Radiation source: fine-focus sealed tube	*R*_int_ = 0.025
graphite	θ_max_ = 25.4°, θ_min_ = 1.4°
ω/2θ scans	*h* = 0→35
Absorption correction: ψ scan (North *et al.*, 1968)	*k* = 0→17
*T*_min_ = 0.936, *T*_max_ = 0.978	*l* = −9→9
3108 measured reflections	3 standard reflections every 200 reflections
3050 independent reflections	intensity decay: 1%

### Refinement

**Table:**

Refinement on *F*^2^	Primary atom site location: structure-invariant direct methods
Least-squares matrix: full	Secondary atom site location: difference Fourier map
*R*[*F*^2^ > 2σ(*F*^2^)] = 0.063	Hydrogen site location: inferred from neighbouring sites
*wR*(*F*^2^) = 0.176	H atoms treated by a mixture of independent and constrained refinement
*S* = 1.00	*w* = 1/[σ^2^(*F*_o_^2^) + (0.098*P*)^2^] where *P* = (*F*_o_^2^ + 2*F*_c_^2^)/3
3050 reflections	(Δ/σ)_max_ = 0.001
228 parameters	Δρ_max_ = 0.41 e Å^−^^3^
0 restraints	Δρ_min_ = −0.28 e Å^−^^3^

### Special details

**Table:**

Geometry. All e.s.d.'s (except the e.s.d. in the dihedral angle between two l.s. planes) are estimated using the full covariance matrix. The cell e.s.d.'s are taken into account individually in the estimation of e.s.d.'s in distances, angles and torsion angles; correlations between e.s.d.'s in cell parameters are only used when they are defined by crystal symmetry. An approximate (isotropic) treatment of cell e.s.d.'s is used for estimating e.s.d.'s involving l.s. planes.
Refinement. Refinement of *F*^2^ against ALL reflections. The weighted *R*-factor *wR* and goodness of fit *S* are based on *F*^2^, conventional *R*-factors *R* are based on *F*, with *F* set to zero for negative *F*^2^. The threshold expression of *F*^2^ > σ(*F*^2^) is used only for calculating *R*-factors(gt) *etc*. and is not relevant to the choice of reflections for refinement. *R*-factors based on *F*^2^ are statistically about twice as large as those based on *F*, and *R*- factors based on ALL data will be even larger.

### Fractional atomic coordinates and isotropic or equivalent isotropic displacement parameters (Å^2^)

**Table:**

	*x*	*y*	*z*	*U*_iso_*/*U*_eq_	
C1	0.05152 (13)	0.3419 (3)	0.6081 (4)	0.0711 (11)	
H1	0.0270	0.3110	0.6471	0.085*	
C2	0.04737 (17)	0.3882 (4)	0.4474 (5)	0.0932 (16)	
H2	0.0195	0.3907	0.3827	0.112*	
C3	0.08311 (19)	0.4293 (3)	0.3852 (5)	0.0898 (14)	
H3	0.0800	0.4570	0.2755	0.108*	
C4	0.12303 (15)	0.4305 (3)	0.4795 (4)	0.0698 (11)	
H4	0.1472	0.4607	0.4363	0.084*	
C5	0.12879 (12)	0.3875 (2)	0.6401 (4)	0.0525 (9)	
C6	0.09271 (11)	0.3426 (2)	0.7089 (4)	0.0490 (8)	
C7	0.09536 (10)	0.2968 (2)	0.8806 (4)	0.0441 (7)	
C8	0.10712 (11)	0.2309 (3)	1.1613 (5)	0.0540 (9)	
C9	0.16054 (10)	0.2020 (2)	1.4126 (4)	0.0438 (7)	
C10	0.16644 (9)	0.1570 (2)	1.5889 (3)	0.0385 (7)	
C11	0.20815 (11)	0.1655 (2)	1.6793 (4)	0.0527 (8)	
H11	0.2302	0.1989	1.6288	0.063*	
C12	0.21841 (13)	0.1272 (3)	1.8389 (5)	0.0630 (10)	
H12	0.2469	0.1350	1.8966	0.076*	
C13	0.18718 (14)	0.0778 (3)	1.9137 (5)	0.0707 (11)	
H13	0.1945	0.0503	2.0220	0.085*	
C14	0.14490 (13)	0.0674 (2)	1.8324 (4)	0.0597 (10)	
H14	0.1235	0.0336	1.8861	0.072*	
C15	0.13365 (10)	0.1075 (2)	1.6690 (4)	0.0419 (7)	
C16	0.20649 (14)	0.4248 (3)	0.6754 (6)	0.1006 (16)	
H16A	0.2114	0.3935	0.5687	0.151*	
H16B	0.2323	0.4173	0.7582	0.151*	
H16C	0.2017	0.4891	0.6517	0.151*	
C17	0.05776 (12)	0.0530 (3)	1.6602 (5)	0.0806 (13)	
H17A	0.0673	−0.0086	1.6895	0.121*	
H17B	0.0310	0.0510	1.5811	0.121*	
H17C	0.0517	0.0851	1.7649	0.121*	
N1	0.06100 (9)	0.2548 (2)	0.9303 (3)	0.0558 (8)	
N2	0.06703 (10)	0.2169 (2)	1.0882 (4)	0.0720 (9)	
O1	0.16829 (8)	0.38751 (17)	0.7453 (3)	0.0615 (7)	
O2	0.11766 (6)	0.19761 (15)	1.3039 (3)	0.0518 (6)	
O3	0.19112 (7)	0.24147 (19)	1.3498 (3)	0.0691 (8)	
O4	0.09264 (7)	0.10003 (17)	1.5783 (3)	0.0561 (6)	
S	0.14064 (3)	0.29401 (7)	1.03358 (11)	0.0554 (3)	
O1W	0.0000	0.1206 (3)	0.2500	0.1159 (19)	
H1W	0.019 (3)	0.159 (6)	0.167 (12)	0.40 (7)*	

### Atomic displacement parameters (Å^2^)

**Table:**

	*U*^11^	*U*^22^	*U*^33^	*U*^12^	*U*^13^	*U*^23^
C1	0.073 (3)	0.091 (3)	0.048 (2)	0.003 (2)	0.0013 (19)	0.009 (2)
C2	0.102 (4)	0.126 (4)	0.048 (2)	0.029 (3)	−0.017 (2)	0.006 (3)
C3	0.134 (4)	0.093 (4)	0.043 (2)	0.019 (3)	0.009 (3)	0.021 (2)
C4	0.120 (3)	0.055 (2)	0.0386 (19)	−0.004 (2)	0.028 (2)	0.0050 (17)
C5	0.077 (2)	0.045 (2)	0.0388 (17)	−0.0061 (17)	0.0212 (17)	0.0022 (15)
C6	0.058 (2)	0.058 (2)	0.0316 (15)	−0.0035 (16)	0.0110 (14)	0.0026 (15)
C7	0.0462 (17)	0.0501 (19)	0.0380 (16)	−0.0056 (15)	0.0142 (13)	0.0003 (14)
C8	0.0469 (19)	0.061 (2)	0.056 (2)	−0.0099 (17)	0.0134 (16)	−0.0026 (18)
C9	0.0421 (16)	0.0516 (19)	0.0399 (16)	−0.0055 (15)	0.0158 (13)	0.0057 (15)
C10	0.0419 (16)	0.0449 (18)	0.0302 (14)	−0.0043 (14)	0.0117 (12)	0.0022 (13)
C11	0.0518 (19)	0.063 (2)	0.0444 (18)	0.0056 (17)	0.0107 (15)	0.0064 (16)
C12	0.060 (2)	0.075 (3)	0.053 (2)	0.014 (2)	−0.0012 (18)	0.0072 (19)
C13	0.094 (3)	0.069 (3)	0.048 (2)	0.013 (2)	−0.001 (2)	0.0129 (19)
C14	0.087 (3)	0.052 (2)	0.0433 (18)	−0.007 (2)	0.0264 (19)	0.0063 (16)
C15	0.0541 (17)	0.0398 (17)	0.0337 (15)	0.0006 (15)	0.0149 (14)	−0.0012 (13)
C16	0.099 (3)	0.110 (4)	0.100 (3)	−0.052 (3)	0.049 (3)	−0.003 (3)
C17	0.071 (2)	0.099 (3)	0.077 (3)	−0.038 (2)	0.031 (2)	−0.006 (2)
N1	0.0557 (17)	0.074 (2)	0.0391 (15)	−0.0111 (15)	0.0085 (13)	0.0099 (14)
N2	0.068 (2)	0.090 (3)	0.0583 (19)	−0.0133 (19)	0.0061 (16)	0.0060 (18)
O1	0.0687 (16)	0.0657 (17)	0.0538 (14)	−0.0200 (13)	0.0250 (13)	0.0021 (12)
O2	0.0355 (11)	0.0463 (13)	0.0756 (16)	−0.0102 (10)	0.0167 (11)	−0.0032 (12)
O3	0.0611 (15)	0.100 (2)	0.0479 (14)	−0.0212 (14)	0.0167 (11)	0.0271 (13)
O4	0.0523 (13)	0.0682 (16)	0.0502 (13)	−0.0190 (12)	0.0183 (11)	0.0020 (11)
S	0.0537 (5)	0.0621 (6)	0.0523 (5)	−0.0108 (4)	0.0158 (4)	0.0026 (4)
O1W	0.083 (3)	0.086 (3)	0.187 (6)	0.000	0.060 (3)	0.000

### Geometric parameters (Å, °)

**Table:**

C1—C6	1.392 (5)	C10—C15	1.401 (4)
C1—C2	1.401 (5)	C11—C12	1.355 (4)
C1—H1	0.9300	C11—H11	0.9300
C2—C3	1.347 (6)	C12—C13	1.346 (5)
C2—H2	0.9300	C12—H12	0.9300
C3—C4	1.338 (5)	C13—C14	1.365 (5)
C3—H3	0.9300	C13—H13	0.9300
C4—C5	1.378 (5)	C14—C15	1.396 (4)
C4—H4	0.9300	C14—H14	0.9300
C5—O1	1.367 (4)	C15—O4	1.357 (4)
C5—C6	1.403 (4)	C16—O1	1.412 (4)
C6—C7	1.472 (4)	C16—H16A	0.9600
C7—N1	1.282 (4)	C16—H16B	0.9600
C7—S	1.709 (3)	C16—H16C	0.9600
C8—O2	1.212 (4)	C17—O4	1.437 (4)
C8—N2	1.291 (4)	C17—H17A	0.9600
C8—S	1.726 (4)	C17—H17B	0.9600
C9—O3	1.214 (3)	C17—H17C	0.9600
C9—O2	1.465 (4)	N1—N2	1.328 (4)
C9—C10	1.499 (4)	O1W—H1W	1.05 (8)
C10—C11	1.375 (4)		
			
C6—C1—C2	119.2 (4)	C10—C11—H11	118.7
C6—C1—H1	120.4	C13—C12—C11	119.7 (4)
C2—C1—H1	120.4	C13—C12—H12	120.1
C3—C2—C1	121.0 (4)	C11—C12—H12	120.1
C3—C2—H2	119.5	C12—C13—C14	120.8 (3)
C1—C2—H2	119.5	C12—C13—H13	119.6
C4—C3—C2	120.7 (4)	C14—C13—H13	119.6
C4—C3—H3	119.7	C13—C14—C15	120.1 (3)
C2—C3—H3	119.7	C13—C14—H14	119.9
C3—C4—C5	120.7 (4)	C15—C14—H14	119.9
C3—C4—H4	119.6	O4—C15—C14	124.1 (3)
C5—C4—H4	119.6	O4—C15—C10	116.8 (3)
O1—C5—C4	124.0 (3)	C14—C15—C10	119.2 (3)
O1—C5—C6	115.4 (3)	O1—C16—H16A	109.5
C4—C5—C6	120.6 (4)	O1—C16—H16B	109.5
C1—C6—C5	117.7 (3)	H16A—C16—H16B	109.5
C1—C6—C7	117.8 (3)	O1—C16—H16C	109.5
C5—C6—C7	124.5 (3)	H16A—C16—H16C	109.5
N1—C7—C6	120.2 (3)	H16B—C16—H16C	109.5
N1—C7—S	112.9 (2)	O4—C17—H17A	109.5
C6—C7—S	126.9 (2)	O4—C17—H17B	109.5
O2—C8—N2	118.9 (3)	H17A—C17—H17B	109.5
O2—C8—S	127.5 (3)	O4—C17—H17C	109.5
N2—C8—S	113.6 (3)	H17A—C17—H17C	109.5
O3—C9—O2	116.4 (3)	H17B—C17—H17C	109.5
O3—C9—C10	122.2 (3)	C7—N1—N2	115.0 (3)
O2—C9—C10	121.3 (2)	C8—N2—N1	112.0 (3)
C11—C10—C15	117.5 (3)	C5—O1—C16	117.3 (3)
C11—C10—C9	116.3 (3)	C8—O2—C9	129.7 (3)
C15—C10—C9	126.2 (3)	C15—O4—C17	118.0 (3)
C12—C11—C10	122.6 (3)	C7—S—C8	86.50 (16)
C12—C11—H11	118.7		
			
C6—C1—C2—C3	3.4 (7)	C13—C14—C15—O4	179.4 (3)
C1—C2—C3—C4	−3.2 (8)	C13—C14—C15—C10	0.9 (5)
C2—C3—C4—C5	1.9 (7)	C11—C10—C15—O4	179.8 (3)
C3—C4—C5—O1	−178.6 (4)	C9—C10—C15—O4	−0.4 (4)
C3—C4—C5—C6	−0.8 (6)	C11—C10—C15—C14	−1.6 (4)
C2—C1—C6—C5	−2.3 (5)	C9—C10—C15—C14	178.2 (3)
C2—C1—C6—C7	177.3 (3)	C6—C7—N1—N2	−179.3 (3)
O1—C5—C6—C1	179.0 (3)	S—C7—N1—N2	−0.5 (4)
C4—C5—C6—C1	1.0 (5)	O2—C8—N2—N1	−177.8 (3)
O1—C5—C6—C7	−0.5 (5)	S—C8—N2—N1	−0.1 (4)
C4—C5—C6—C7	−178.4 (3)	C7—N1—N2—C8	0.4 (5)
C1—C6—C7—N1	2.8 (5)	C4—C5—O1—C16	−7.5 (5)
C5—C6—C7—N1	−177.7 (3)	C6—C5—O1—C16	174.6 (3)
C1—C6—C7—S	−175.8 (3)	N2—C8—O2—C9	179.1 (3)
C5—C6—C7—S	3.7 (5)	S—C8—O2—C9	1.8 (5)
O3—C9—C10—C11	2.2 (5)	O3—C9—O2—C8	−3.4 (5)
O2—C9—C10—C11	179.9 (3)	C10—C9—O2—C8	178.8 (3)
O3—C9—C10—C15	−177.7 (3)	C14—C15—O4—C17	4.1 (5)
O2—C9—C10—C15	0.1 (5)	C10—C15—O4—C17	−177.4 (3)
C15—C10—C11—C12	0.9 (5)	N1—C7—S—C8	0.4 (3)
C9—C10—C11—C12	−179.0 (3)	C6—C7—S—C8	179.1 (3)
C10—C11—C12—C13	0.7 (6)	O2—C8—S—C7	177.3 (4)
C11—C12—C13—C14	−1.4 (6)	N2—C8—S—C7	−0.2 (3)
C12—C13—C14—C15	0.7 (6)		

### Hydrogen-bond geometry (Å, °)

**Table:**

*D*—H···*A*	*D*—H	H···*A*	*D*···*A*	*D*—H···*A*
O1W—H1W···N2^i^	1.05 (9)	1.81 (9)	2.821 (4)	159 (7)
C11—H11···O3^ii^	0.93	2.50	3.324 (4)	148

Symmetry codes: (i) *x*, *y*, *z*−1; (ii) −*x*+1/2, −*y*+1/2, −*z*+3.
